# Cement loaded with high-dose gentamicin and clindamycin does not reduce the risk of subsequent infection after aseptic total hip or knee revision arthroplasty: a preliminary study

**DOI:** 10.1186/s10195-024-00775-1

**Published:** 2024-07-24

**Authors:** Ceyran Hamoudi, Marie Hamon, Aurélie Reiter-Schatz, Pierre-Antoine Debordes, Jeannot Gaudias, Cécile Rondé-Oustau, Jean-Yves Jenny

**Affiliations:** 1 Department of Orthopaedic Surgery, Hôpitaux Universitaires de Strasbourg, Strasbourg, France; 2 Department of Pharmacy, CHU de Caen, Caen, France; 3 Clinique Sainte-Odile, Groupe Elsan, Haguenau, France; 4 Impulse-Ortho, 3 Rue de la Redoute, F-67500 Haguenau, France

**Keywords:** Dual antibiotic loaded bone cement, Aseptic revision, Periprosthetic joint infection, Surgical site infection

## Abstract

**Purpose:**

The aim of this study was to quantify the prophylactic effect of high-dose gentamicin and clindamycin antibiotic-loaded bone cement (ALBC) during revision total hip (rTHA) or knee (rTKA) arthroplasty for aseptic reasons. The hypothesis was that the raw surgical site infection (SSI) rate is lower when this particular cement is used in comparison with cement loaded with standard-dose gentamicin during rTHA or rTKA for aseptic reasons.

**Methods:**

This retrospective study included 290 consecutive patients undergoing aseptic rTHA or rTKA. Two consecutive cohorts were defined: the first (control group) involved 145 patients where ALBC with gentamicin only was used; the second (study group) involved 145 patients where ALBC with high-dose gentamicin and clindamycin was used. The primary endpoint was the raw SSI rate after 24 months.

**Results:**

The raw SSI rate was 8/145 (6%) in the control group and 13/145 (9%) in the study group (odds ratio 0.62, *p* = 0.26). There was a significant impact of the presence of any risk factor on the SSI rate (15/100 versus 6/169, odds ratio = 4.25, *p* = 0.002), but no significant impact of any individual risk factor. No complication or side effect related to ALBC was observed in either group.

**Conclusion:**

These results do not support the routine use of gentamicin and clindamycin ALBC for fixation of revision implants after rTHA and rTKA for aseptic reasons.

## Introduction

Aseptic total hip (rTHA) and knee (rTKA) revision arthroplasty is associated with an increased risk of developing periprosthetic joint infections (PJI) compared with primary arthroplasty [1]. Strategies to prevent this complication include prophylactic antibiotics, meticulous skin preparation and surgical site irrigation [2]. When cemented implants are used, antibiotic-loaded bone cement (ALBC) may help to prevent infection [3]. The primary goals of ALBC are to release locally high antibiotic concentrations above the inhibitory level [4] and to prevent bacterial adhesion to the implants [5]. Most ALBC commercially available are loaded with only one antibiotic. The antibiotic most used in commercial preparations is gentamicin [6], although other antibiotics are available, namely vancomycin or tobramycin.

The optimal place of ALBC in primary hip and knee replacement remains debated [7]. Its use during revision arthroplasty is less documented [8]. More recently, bone cement loaded with two antibiotics has been developed to improve efficacy. For instance, cement loaded with high-dose gentamicin and clindamycin, also called dual ALBC (DALBC), proved to be effective for prophylactic use in aseptic knee revision [9] and had a positive impact on infection after one-stage revision arthroplasty for periprosthetic joint infection (PJI) [10].

The aim of this study was to quantify the prophylactic effect of high-dose DALBC during rTHA or rTKA for aseptic reasons. The hypothesis was that the raw surgical site infection (SSI) rate is lower when this particular cement is used in comparison to gentamicin single ALBC (SALBC) during rTHA or rTKA for aseptic reasons.

## Material and methods

This retrospective, single-center, observational study was conducted in compliance with the recommendations of the Helsinki declaration. It was approved by the Ethics Committee of the Strasbourg University Hospitals (file no. 2019-26), and all patients gave their written consent. All patients who were operated by two experienced surgeons for aseptic rTHA or rTKA between 1 April 2015 and 31 December 2020 were eligible. Excluded were patients who declined to participate in this study, patients with a suspected periprosthetic infection according to the MusculoSkeletal Infection Society (MSIS) [11], or patients who were minors or legally incompetent. The following preoperative data were compiled: age, gender, weight, height, body mass index (BMI), American Society of Anesthesiologists (ASA) classification [12], National Nosocomial Infections Surveillance (NNIS) risk index [13], and any medical conditions that might contribute to infection (i.e., diabetes, rheumatoid disease, smoking, current immunosuppressive treatment, and obesity).

The surgical procedures were standardized: anterolateral approach to the hip or anteromedial approach to the knee, partial or complete removal of implants and any existing cement at the site of removal, and reconstruction with cemented implants and bone allograft, as needed. The administration of antibiotics was also standardized: routine conventional antimicrobial prophylaxis [cefazolin (Mylan, Saint-Priest, France) 2 or 4 g depending on body weight] during anesthetic induction.

The patients were divided into two consecutive cohorts based on the type of cement used: SALBC with 0.5 g of gentamicin (Palacos R + G ^®^, Heraeus, Hanau, FRG) in the first cohort (1 April 2015 to 15 February 2018; control group) and DALBC with 1 g of gentamicin and 1 g of clindamycin (Copal G + C^®^, Heraeus, Hanau, FRG) in the second cohort (16 February 2018 to 31 December 2020; study group).

All patients were followed for a minimum of 24 months. A surgical site infection (SSI) was defined according to the MSIS definition [11]. The occurrence of an SSI was recorded and classified as early (< 3 months after index revision) or late (> 3 months after index revision). The rate of repeat surgery for SSI was monitored. The susceptibility of the bacteria found in the SSI to gentamycin and clindamycin was noted. Any complication or side effect during the postoperative survey was documented, and its relationship to the bone cement was analyzed.

The primary criterion was the raw SSI rate at the 2-year follow-up. The secondary criteria were:

- the cumulative SSI rate within 2 years of the revision surgery
- the number of repeat surgeries for SSI
- the susceptibility of the micro-organisms isolated during subsequent SSI treatment
- the influence of preoperative risk factors on the SSI rate and the impact of ABLC.

The collected data were compared between groups with a chi squared test or a Fisher’s exact test and calculation of the odds ratio for qualitative data and by Student’s *t* test or the Mann–Whitney *U* test for quantitative data. The effect of prognostic factors was analyzed in the same manner. The cumulative rate of cases without SSI was plotted according to Kaplan–Meier, and the survival rates were compared in the two groups with a log-rank test. All statistical tests were done with a 5% threshold.

Several recent publications have reported that the SSI rate after aseptic rTHA or rTKA ranges from 2% to 15% [14–17]. The sample size was calculated on the basis of the following assumptions: comparison of two groups, raw SSI rate in control group of 10%, detection of a reduced raw SSI rate of 5% in the study group, alpha risk of 0.05, and beta risk of 0.20. According to this calculation, a minimum of 138 patients per group was needed.

## Results

A total of 290 patients’ records were included. There were 141 men (49%) and 149 women (51%) with a mean age of 69 ± 11 years and a mean body mass index (BMI) of 29.3 ± 6.0 kg/m^2^. Seventeen were classified as American Society of Anesthesiologists (ASA) status 1 (6%), 166 as ASA 2 (57%), and 107 as ASA 3 (37%). A total of 166 patients had a risk index of National Nosocomial Infection Surveillance (NNIS) index 0 (57%), 100 were NNIS 1 (34%), and 24 were NNIS 2 (8%). The hip was operated on in 142 patients (49%) and the knee was operated on in 148 patients (51%). There were 145 patients in the study group and 145 patients in the control group. There was no significant difference in the preoperative parameters between the two groups, except that the control group contained younger patients and had more knees than hips (Table 1). The reason for revision is reported in Table 2.

**Table 1:** Characteristics of the study population

	Control group (*n* = 145)	Study group (*n* = 145)	Significance(*P* value)
Age (years), mean ± standard deviation	66 ± 11	71 ± 11	0.001*
Gender (men/woman)	72/73	69/75	0.77
BMI (kg/m^2^), mean ± standard deviation	29.4 ± 6.5	29.0 ± 5.6	0.58
ASA score (1/2/3)	9/78/58	8/88/49	0.49
NNIS risk index (0/1/2/3)	76/53/16/0	90/47/8/0	0.12
Diabetes (yes/no)	31/114	30/115	0.89
Rheumatic disease (yes/no)	14/131	10/135	0.39
Current smoker (yes/no)	28/117	20/125	0.21
Immunosuppression (yes/no)	11/134	6/139	0.21
Obesity with BMI > 30 kg/m^2^ (yes/no)	64/81	58/87	0.48
Any risk factor (yes/no)	63/82	52/93	0.19
Hip/knee	58/87	84/61	0.002*

^*^Denotes a significant difference

**Table 2:** Reason for revision

	Hip (142 cases)	Knee (148 cases)
Aseptic loosening	116	124
Instability	28	14
Acetabular impingement	8	–
Rotational malposition	–	10

**Fig. 1:**
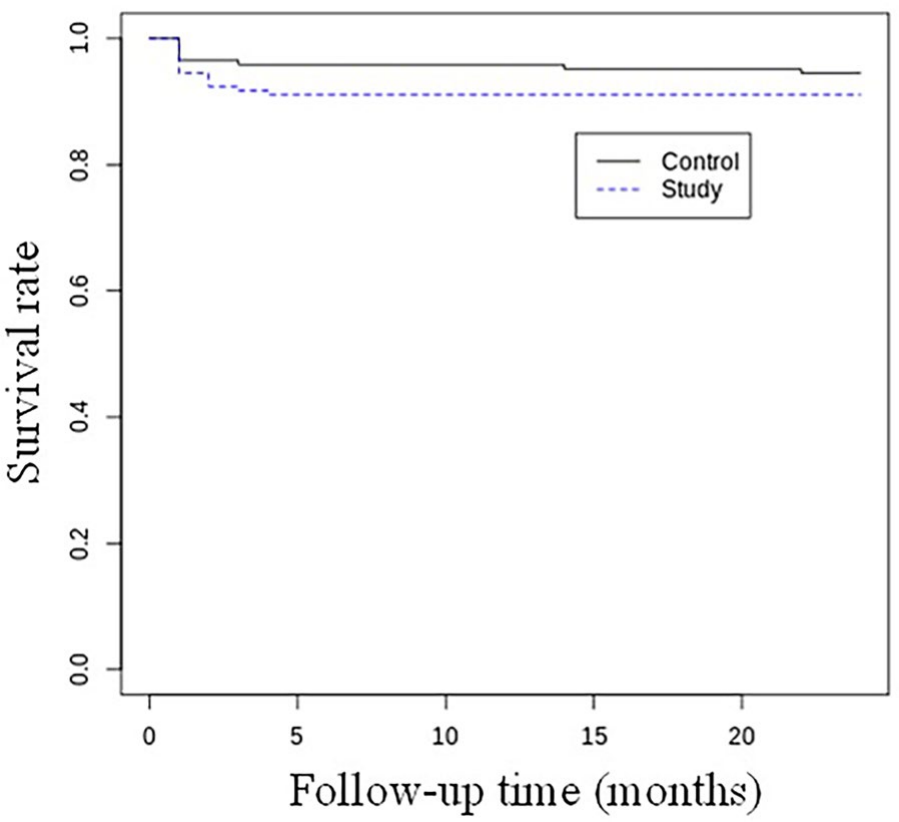
Kaplan–Meier survival curves: cases without SSI

The raw SSI rate was 8/145 (6%) in the control group and 13/145 (9%) in the study group (odds ratio 0.62, *p* = 0.26) (Table 3). The cumulative rate of cases without SSI after 2 years was 94% in the control group and 92% in the study group (Fig. 1) (*p* = 0.25). There was no significant difference between groups in the distribution between early and late SSIs (*p* = 0.06) (Table 4). There was no significant difference between groups in the distribution between hip and knee SSIs (*p* = 0.06) (Table 5).

**Table 3:** Effect of ALBC

	SSI	No SSI	Odds ratio	Significance (*P* value)
Control group (*n* = 145)	116	124	0.62	0.26
Study group (*n* = 145)	28	14

**Table 4:** Time to diagnosis of SSI

	Control group (*n* = 8)	Study group (*n* = 13)	Significance
Early (< 3 months)	6	13	0.13
Late (> 3 months)	2	0	

^*^Denotes a significant difference

**Table 5:** Location of arthroplasty

	Control group (*n* = 145)	Study group (*n* = 13)	Significance
Hip (SSI/no SSI)	1/57	8/76	0.08
Knee (SSI/no SSI)	7/80	5/56	1.00

^*^Denotes a significant difference

All SSI cases had repeat surgeries (Table 6). All cases were ultimately infection-free at the latest follow-up, except one case in the study group that had a persistent knee PJI and received suppressive antibiotic treatment.

**Table 6:** Nature of repeat surgery for SSI

	Control group (*n* = 8)	Study group (*n* = 13)	Significance
Debridement, antibiotics, and implant retention	3	10	0.16
One-stage exchange arthroplasty	5	3	

^*^Denotes a significant difference

The microbiological analysis of the SSI cases is reported in Table 7. In 2 of the 8 cases in the control group and in 6 of the 13 cases in the study group, all the bacteria responsible for the SSI were resistant to gentamycin and clindamycin. In the control group, the unknown susceptibility was due to a concomitant infection with *Candida albicans* and *Enterococcus faecalis* in one case and a concomitant infection with *Candida albicans* and *Klebsiella varicola* in one case. In the study group, the unknown susceptibility to gentamycin and clindamycin was due to *Candida albicans* infection in one case, *Finegoldia Magna* infection in one case, *Enterococcus faecalis* infection in three cases, and a concomitant infection with *Klebsiella aerogenes* and *Enterococcus faecalis* in one case.

**Table 7:** Microbiological analysis of SSI

	Control group (*n* = 8)	Study group (*n* = 13)	Significance (*P* value)
All bacteria susceptible to gentamicin and clindamycin	2	1	0.53
At least one bacterium resistant to gentamycin	4	8	0.67
At least one bacterium resistant to clindamycin	4	7	1.00
All bacteria resistant to gentamicin and clindamycin	1	3	1.00
Unknown susceptibility	2	6	

^*^Denotes a significant difference

There was a significant impact of the presence of any risk factor on the SSI rate (odds ratio = 4.25, *p* = 0.002) but no significant impact of any individual risk factor on the SSI rate, without any difference between the two groups (Tables 8 and 9).

**Table 8:** Impact of prognostic factors

Effect of any risk factor	SSI	No SSI	Odds ratio	Significance (*p* value)
Any risk factor	15	100	4.25	0.002*
No risk factor	6	169		

Prognostic factor	Control group (*n* = 145)	Study group (*n* = 145)	Odds ratio	Significance (*p* value)
Current smoker: SSI/no SSI	1/27	3/17	4.2	0.75
Non-smoker: SSI/no SSI	7/110	10/115	1.3	0.54
Immunosuppression: SSI/no SSI	3/8	0/6	1.8	0.16
No immunosuppression: SSI/no SSI	5/129	13/126	2.5	0.06
Obesity: SSI/no SSI	5/59	7/51	1.5	0.43
No obesity: SSI/no SSI	3/78	6/81	1.9	0.36

^*^ Denotes a significant difference

**Table 9:** Interaction between risk factors and ALBC

Interaction risk factor/ALBC	Control group	Study group	Odds ratio	Significance (*p* value)
Any risk factor: SSI/no SSI	7/56	8/44	1.45	0.50
No risk factor: SSI/no SSI	1/73	5/88	4.15	0.17
Odds ratio	9.13	3.2		
	0.02*	0.04*		

^*^Denotes a significant difference

No complication or side effect related to ALBC was observed in either group.

## Discussion

This study’s main finding is that the raw SSI rate was not lower when cement loaded with 1 g gentamicin and 1 g clindamycin (DALBC) was used during rTHA or rTKA compared with when cement loaded with a standard dose of 0.5 g gentamicin (SALBC) was used. Furthermore, the cumulative SSI rate within 2 years of the revision surgery, the number of repeat surgeries for SSI, the susceptibility of the microorganisms isolated during subsequent SSI treatment were not different between groups, and no influence of preoperative risk factors on the SSI rate was observed in either group.

Currently, the prophylactic use of ALBC is a common practice in cemented hip and knee arthroplasty. ALBC has shown to be effective in reducing PJI after primary cemented total hip or knee replacement [18, 19]. However, opposite results have also been published [20, 21], thus this issue remains controversial. As the PJI risk is higher after rTHA and rTKA, one might hypothesize that the positive impact of using ALBC will be even higher [8].

There is some rationale for using DALBC instead of SALBC. The larger dose of gentamycin delivered locally, and the addition of clindamycin may improve efficacy. Ensing et al. [22] have showed that DALBC (gentamicin + clindamycin) was more effective against biofilm formation than SALBC (gentamicin only).

Abdelaziz et al. [23] reported that the use of gentamicin and clindamycin DALBC during aseptic revision arthroplasty helped to prevent infection in high-risk patients, but no control group was incorporated into their study. In a retrospective study, Sanz-Ruiz et al. [9] observed no PJI after 103 cases of aseptic rTKA using DALBC (gentamicin + clindamycin), while 6 PJIs were observed after 143 cases of aseptic rTKA using SALBC (gentamicin only). The present study is the first to compare the results of using high-dose DALBC to gentamicin SALBC for preventing PJI after both aseptic rTHA or rTKA. However, it did not show favorable results after prophylactic use of DALBC in PJI prevention after aseptic revisions compared with SALBC. The difference between the two studies might be explained by a selection bias or a longer follow-up.

It could be assumed that this DALBC would be useful in certain subgroups of patients, especially those at high risk of postoperative infection. The present study confirms this hypothesis when all risk factors were grouped together, but not for any individual risk factor. However, it was not designed to answer this question. Other studies with larger numbers of patients could allow us to analyze this point.

There remains a concern about the risk of side-effects after using ALBC. However, a large review study including more than 2 millions cases demonstrated that there was no impact on the side-effect rate when using ALBC in comparison with plain cement [24].

In the current constrained economic situation, there is a concern about the additional costs of using ALBC. However, Sanz-Ruiz et al. reported that ALBC and DALBC was cost-effective in comparison with plain cement, due to the reduction in the infection rate [9, 21].

## Limitation

This study has several limitations. While the data were collected prospectively and consecutively, this study was basically a retrospective one, with all the potential biases inherent in this type of study, even though the comparability of the two groups was established and the differences observed preoperatively did not affect the differences in SSI development. Also, this was a continuous case series with no patient selection for medical reasons, involving two cohorts that were operated consecutively with no alterations in the surgical technique or medical care, particularly the prophylactic antibiotic therapy. The number of patients in these two cohorts is relatively small, but the study’s sample size was calculated beforehand to answer a specific question, thus making the results valid. The included population appears similar to that of larger studies and could actually be considered as representative. The patients in the study group were significantly older than those in the control group; however, it was demonstrated that age alone was not a risk factor for periprosthetic joint infection [25], and we assume that this does lead to a significant bias. The follow-up was insufficient to detect very late infections and may be insufficient for determining the long-term survival of the cement fixation. Lastly, the study was under-powered to perform subgroup analysis.

## Conclusion

The present results do not support the routine use of gentamicin and clindamycin DALBC for the fixation of revision implants after rTHA and rTKA for aseptic reasons. Its use in high-risk patients still needs to be defined.

## Data Availability

Data will not be deposited.
